# Iron-oxide nanoparticle-integrated soft contact lenses: optical optimization, catalytic functionality, and biocompatibility

**DOI:** 10.3389/fmedt.2026.1782878

**Published:** 2026-07-24

**Authors:** Aswathi Ram P, Muhammed Shebeeb, Muhammed Hisham, Sanjana Chandran, Said El Turk, Haider Butt

**Affiliations:** 1 Department of Mechanical and Nuclear Engineering, Khalifa University of Science and Technology, Abu Dhabi, United Arab Emirates; 2 Department of Biomedical Engineering and Biotechnology, Khalifa University of Science and Technology, Abu Dhabi, United Arab Emirates

**Keywords:** contact lens, iron oxide nanocomposite, nanocomposite hydrogel, nanoparticle-incorporated contact lens, nanozyme contact lens

## Abstract

Millions of people suffer from corneal infections and inflammatory conditions, and a contact lens that not only corrects vision but also continuously protects against microbes and inflammation could transform ocular therapeutics. Compared with plasmonic metal nanoparticles, such as silver or gold, iron oxide nanoparticles are well known for their biocompatibility, low toxicity, and low cost. To our knowledge, this work is the first to demonstrate the integration of iron oxide nanoparticles into commercial contact lenses. Nanoparticles were characterized using X-ray diffraction, X-ray photoelectron spectroscopy, scanning electron microscopy and transmission electron microscopy. Optical properties were assessed using UV-Vis spectroscopy and COMSOL simulations. Catalytic activity was evaluated by methylene blue degradation in the presence of H_2_O_2_. Lens's biocompatibility, anti-inflammatory and anti-bacterial properties were investigated using fibroblast cell viability assays and antibacterial testing. X-ray diffraction verified the structure with a lattice parameter of 8.37 Å, while XPS shows that the obtained nanoparticles (NPs) consisted of a mixture of both Fe_3_O_4_/γ-Fe_2_O_3_ NPs. SEM and transmission electron microscope confirmed that the NPs are uniformly dispersed and have an average size of ∼8 nm. UV-vis spectroscopy results showed significant optical transmission after embedding the NPs. The COMSOL simulation data indicated that smaller nanoparticle radii maintain high transparency and larger radii reduce transmission. At higher nanoparticle loading, the lenses exhibited 96.7% methylene blue degradation in the presence of H_2_O_2_ over 2 weeks. Biocompatibility testing showed >70% fibroblast cell viability with normal morphology, along with anti-inflammatory and antibacterial activity against *Staphylococcus aureus*. Findings show the potential of iron-oxide nanoparticle-embedded contact lenses as transparent, catalytically active, and biologically safe platforms for future therapeutics and wearable optics applications.

## 1 Introduction

Contact lenses are widely used vision-correcting devices; however, their long-term wear is frequently associated with microbial keratitis, inflammatory complications, and biofilm formation (1). Conventional eye drops suffer from significant limitations, including rapid tear dilution, short ocular residence time, and poor patient adherence (2, 3). These shortcomings necessitate the development of next-generation contact lenses capable of providing sustained antimicrobial protection while maintaining optical clarity and biocompatibility.

Nanoparticles have often been investigated for their biomedical applications (4–9). Iron oxide nanoparticles are among the most widely used nanomaterials in biomedicine because of their unique and excellent biocompatibility, with antimicrobial activity mediated by their catalytic activity. They have demonstrated significant antibacterial efficacy against biofilm formation on biomaterial surfaces (10). Previously explored nanoparticle systems for contact lenses, such as silver and zinc oxide, have significant limitations, including cytotoxicity and UV-dependent activation (11). Gold nanoparticles (NPs) are safe but expensive and have limited functionalization. Fe_3_O_4_ and Fe_2_O_3_ possess unique catalytic, optical, and magnetic properties that make them attractive for multifunctional systems (12–14). Fe_3_O_4_ nanoparticles catalyze the decomposition of hydrogen peroxide into highly reactive hydroxyl radicals with strong bactericidal effects. Incorporation of these NPs into contact lenses in the present study offers a combination of inherent biocompatibility, catalytic activity, and antimicrobial efficacy, which addresses the key limitations of previously reported nanoparticle-based contact lens systems. In addition, iron oxide NPs have a high specific surface area and porous structure that give them outstanding absorption capacity and water and oxygen permeability, making them ideal for wound dressings and drug delivery, which could be explored in the future. In this study, we developed a Fe_3_O_4_ and Fe_2_O_3_ nanoparticle-embedded soft contact lens synthesized via a controlled co-precipitation method. The supreme objective of this work is to understand how iron oxide nanoparticles influence the optical transparency, catalytic activity, and biocompatibility of soft contact lenses while maintaining properties suitable for ophthalmic use. Following detailed structural, optical, and chemical characterizations, the crystalline phase was identified by X-ray diffraction (XRD), while energy-dispersive X-ray spectroscopy (EDS) and X-ray photoelectron spectroscopy (XPS) were used to determine the elemental composition and oxidation states. Fourier transformed infrared spectrum (FTIR) showed no significant chemical bond formation. To calculate the optical band gap and transparency characteristics of the embedded lenses, UV–vis absorption spectra were analysed, which are crucial for ophthalmic applications. The catalytic activity of the iron oxide nanoparticles was evaluated by measuring of methylene blue (MB) degradation, showcasing their potential for reactive oxygen species (ROS)-mediated antimicrobial and pollutant degradation applications. Progressive degradation of methylene blue absorbance confirmed sustained •OH production by the embedded iron oxide nanoparticles, providing a reliable assessment of catalytic functionality. Biocompatibility and cell viability assays indicated non-toxicity and a favourable interaction with biological tissues, with no acute cytotoxicity under the tested conditions.

We further introduced COMSOL modelling to predict the size-dependent scattering and transmission behaviour. The Comsol outputs (e.g., transmittance vs. NP radius at specific wavelengths, field intensity maps) add to the novelty of this work. To know knowledge, no study to date has used COMSOL finite-element simulation to explicitly model the effect of iron oxide nanoparticle radius on visible-light transmission in a contact lens matrix. The combined experimental and computational results define the optimum NP size and loading for a magnetizable, self-sterilizing lens. Notably, while we observed that the NP-loaded lenses were responsive to magnets, we did not exploit this property in the present work. The inherent superparamagnetism of Fe_3_O_4_ and Fe_2_O_3_ could enable future ophthalmic functions—for example, magnetically guided drug delivery, magnetic resonance imaging contrast, or stimuli-responsive optics. We recommend follow-up experiments and *in vivo* studies to explore these possibilities.

## 2 Experimental section

### 2.1 Materials and methods

#### 2.1.1 Materials

Acuvue Oasis (Senofilcon A) contact lenses were obtained from Acuvue, Johnson & Johnson. Neonatal human dermal fibroblasts (Thermo Fisher Scientific, C0045C) were used for cell viability and inflammatory response studies. TRIzol reagent (Invitrogen) and trypan blue dye (Sigma-Aldrich) were employed for RNA extraction and cell viability assays, respectively. Foetal bovine serum (FBS), minimum essential medium (MEM), Dulbecco's phosphate-buffered saline, and trypsin–ethylenediaminetetraacetic acid (trypsin–EDTA) were obtained from Gibco. The bacterial strains *Pseudomonas aeruginosa* and *Staphylococcus aureus* were procured from AstraGene, and Luria–Bertani (LB) broth powder was purchased from Sigma-Aldrich. Iron(III) nitrate nonahydrate, iron(II) chloride tetrahydrate sodium hydroxide, and hydrogen peroxide were obtained from Sigma-Aldrich.

### 2.2 Iron-oxide nanoparticle-incorporated lens synthesis

**Figure 1:**
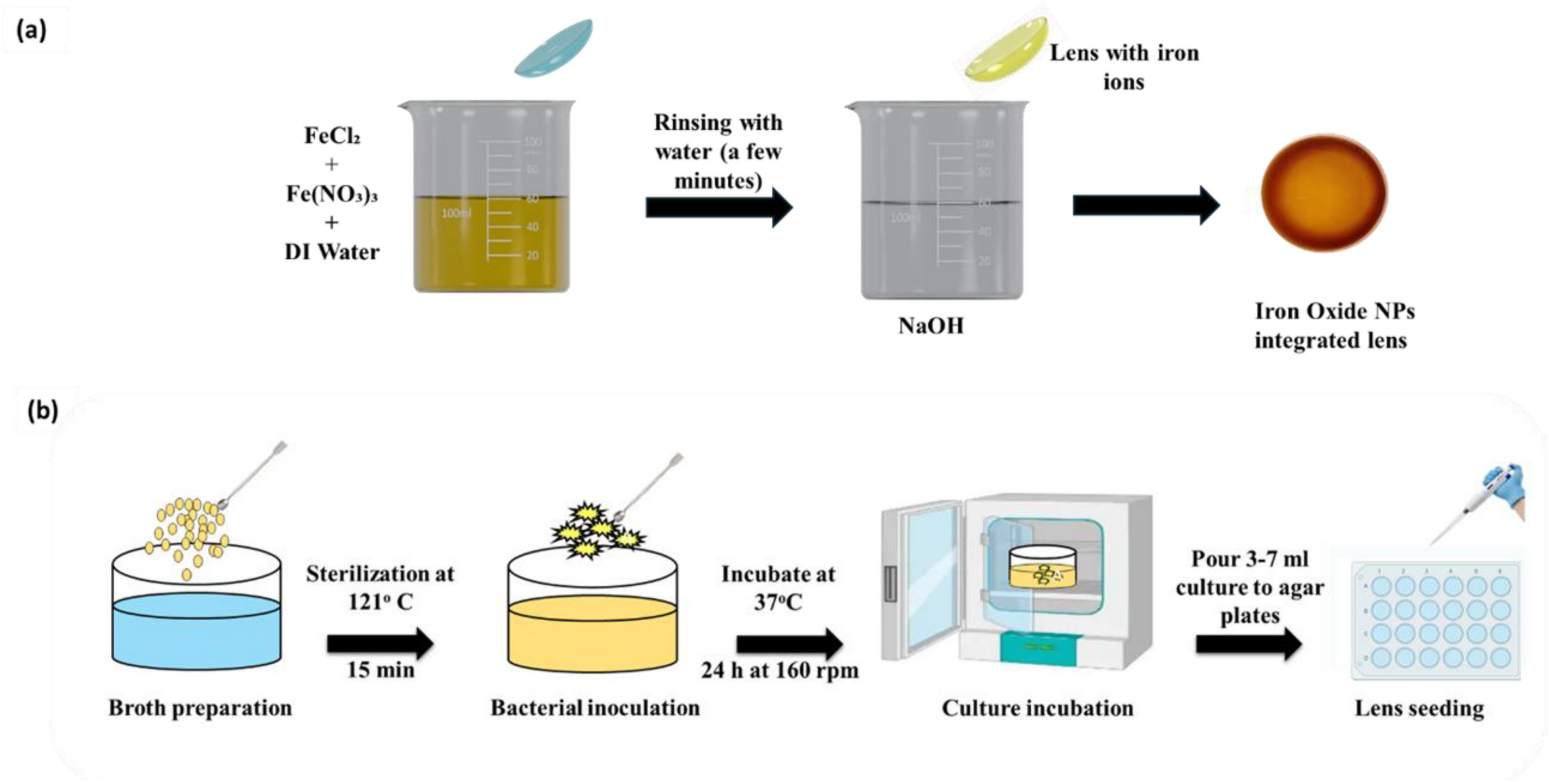
**(a)** Synthesis of iron oxide NPs embedded in contact lenses. **(b)** Bacterial culture preparation and lens seeding (15, 16).

The in situ precipitation method was used to incorporate nanoparticles into the contact lens. Two different approaches were adopted to control the nanoparticle concentration and distribution within the lens matrix. The schematic of the synthesis is shown in Figure 1.

#### 2.2.1 Multiple immersion cycles

In this approach, the contact lenses were immersed in the iron precursor solution [iron(II) chloride tetrahydrate, amount in grams equivalent to get 0.1 M Fe^2+^, and iron(III) nitrate nonahydrate, amount in grams equivalent to obtain 0.2 M Fe^3+^] for a fixed duration of 30 min to ensure the diffusion of iron ions into the lens matrix. After 30 min, the lenses were gently rinsed with deionized (DI) water and transferred into 0.5 M NaOH solution, where the hydroxide ions reacted with the absorbed iron ions, leading to *in situ* formation of iron oxide nanoparticles. The procedure was conducted at room temperature. The pH was not controlled or systematically studied in the present study. The same process was repeated for many cycles by alternatively immersing the lenses in the iron precursor and NaOH solutions to increase the concentration. The information about the concentrations are given in Supplementary Table S1.

#### 2.2.2 Variation in precursor concentration

In this approach, the same *in situ* precipitation method was followed, but the molarity of the iron precursor solutions was varied. Contact lenses (one per lens) were immersed in precursor solutions of different concentrations, followed by treatment with NaOH for nanoparticle synthesis. The information about the concentrations is given in Supplementary Table S2.

### 2.3 Catalytic activity testing of iron oxide nanoparticles

Preparation of MB solution: A 25 mg/L methylene blue solution is made by dissolving 11.25 mg of dye in 225 mL of distilled water. From this stock solution, 75 mL was mixed with 75 mL of hydrogen peroxide (H_2_O_2_). The resulting mixture was used for the photocatalytic degradation study, in which lenses embedded with nanoparticles of varying concentrations were placed in a well plate. The concentration of each lens and the sample code is given in Table 1. The samples were monitored regularly over a period of 2 weeks to observe the degradation behaviour of the methylene blue solution. The percentage of MB dye degradation was calculated using the Equation 1:

$$\mathrm{Degradation}(\text{\%})=\frac{Ci-\;Ct}{Ci}\times 100$$ (1)

**Table 1:** Concentration of each lens and the sample code.

Sample code	Concentration (0.1 M Fe^2+^+ 0.2 M Fe^3+^)	Sample code	Concentration	Number of cycles
C_1_	Cycle 1	C_8_	0.1 M Fe^2+^ + 0.2 M Fe^3+^	1 cycle
C_2_	Cycle 2	C_9_	0.2 M Fe^2+^ + 0.4 M Fe^3+^	1 cycle
C_3_	Cycle 3	C_10_	0.3 M Fe^2+^ + 0.6 M Fe^3+^	1 cycle
C_4_	Cycle 4	C_11_	0.4 M Fe^2+^ + 0.8 M Fe^3+^	1 cycle
C_5_	Cycle 5	C_12_	0.5 M Fe^2+^ + 1.0 M Fe^3+^	1 cycle
C_6_	Cycle 6	C_14_	0.5 M Fe^2+^ + 1.0 M Fe^3+^	6 cycles

### 2.4 Electromagnetic modelling

**Figure 2:**
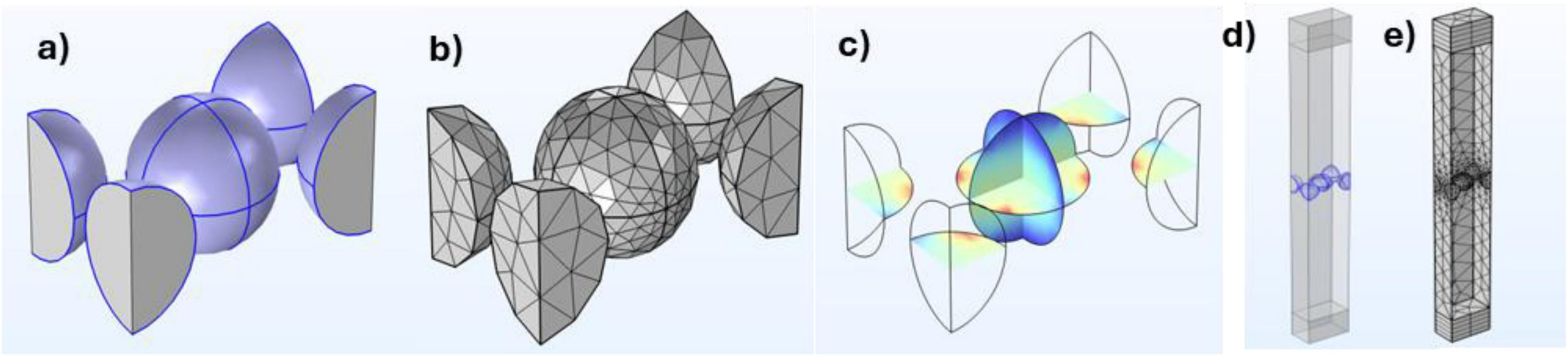
**(a)** Geometry of the nanoparticle cluster showing iron oxide nanoparticles arranged within the unit cell. **(b)** Finite-element mesh generated for the nanoparticle domains. **(c)** Simulated electric field distribution around the nanoparticles. **(d)** Full simulation domain including the unit cell and PML layers. **(e)** Complete volumetric mesh of the entire model (unit cell + PML + nanoparticles).

For electromagnetic modelling, the infinitely extended 2D arrays were represented using a unit-cell model, with boundary conditions applied along the lateral surfaces. The model represents an idealized ordered nanoparticle arrangement and was used to evaluate the optical response. The incoming electromagnetic waves were introduced from the top boundary, while the bottom boundary served as the wave exit to obtain complete wave distribution. Perfectly matched layers (PML) were placed at both the upper and lower boundaries to absorb outgoing waves and prevent wave reflections. To validate the accuracy of the periodic approximation, the mesh density and computational domain size were systematically varied, further confirming that the numerical values are independent of these parameters (15). Mesh convergence was assessed by systematically refining the mesh until no appreciable change in the transmission was observed with additional refinement. Higher-order effects arising from random clustering were not modelled. The geometry, finite-element mesh, electric field distribution, simulation domain, and complete volumetric mesh of the computational model are shown in Figure 2. Details of material dielectric constants (*n*, *k*) used for Fe, Fe_3_O_4_, and Fe_2_O_3_ across the wavelength range are referred from the technical report of optical constants (16).

Unit cell dimensions were calculated as follows:

Width=(2*radius+d)*cos(pi/6)*2,Depth=(2*radius+d),Height=30*radius

where *d* is the interparticle distance.

### 2.5 Cell viability assessment

Cytocompatibility of the iron-oxide nanoparticle-coated contact lenses was evaluated using the trypan blue exclusion assay. Neonatal human dermal fibroblast (HDFn) cells (Thermo Fisher Scientific, C0045C) were cultured in MEM (Gibco) supplemented with 20% FBS at 37°C in a humidified 5% CO_2_ incubator. Cells were seeded in a 12-well plate at a density of 1.5 × 10^5^ cells mL^−1^ and allowed to adhere for 24 h prior to lens exposure. The NP-coated lenses were pre-sterilized using an autoclave. They were rinsed thoroughly with serum-free MEM to remove unbound particles and placed in direct contact with the cell monolayer in the wells for 24 h. Wells with commercially available clear lenses were included, and wells without lenses served as the control group. After 24 h of incubation, the lenses were removed, and the cells were detached using 0.025% trypsin–EDTA and mixed 1:1 with trypan blue solution (Sigma-Aldrich). The cells were counted using the Invitrogen Countess 3 FL automatic cell counter after the 24-h incubation. For each lens group, cells from a minimum of two wells were collected independently and assayed. In addition, cell counts for each well were measured in duplicate using the assay, and statistical outliers were identified and excluded prior to analysis. The percentage of viable cells relative to the total number of cells was used to indicate cell viability. Statistical analysis was performed using one-way ANOVA (GraphPad Prism 9), with *p* < 0.05 considered statistically significant (17, 18).

### 2.6 Anti-inflammatory gene expression analysis

The inflammatory response of fibroblasts following exposure to nanoparticle-coated lenses was assessed by real-time quantitative polymerase chain reaction (RT-qPCR) targeting the cytokine genes tumour necrosis factor-*α* (TNF-*α*), interleukin-6 (IL-6), and interleukin-1β (IL-1β). As described in the previous section, HDFn cells were cultured and treated. After 24 h of incubation with the lenses (three wells per group), according to the manufacturer's protocol, total RNA was extracted using TRIzol reagent (Invitrogen). Following removal of lenses after 24 h, cells from three wells were pooled to obtain sufficient RNA. RNA concentration and purity were verified spectrophotometrically (NanoDrop 2000, Thermo Scientific). cDNA was synthesized using the High-Capacity cDNA Reverse Transcription Kit (Applied Biosystems). qPCR amplification was performed using Taq Pro Universal SYBR qPCR Master Mix (Vazyme) on a MicPCR system (BioMolecular Systems). Table 2 lists the primer sequences that were used. The endogenous reference gene was GADPH. Relative gene expression levels were computed using the 2^−ΔCt^ method and normalized to the untreated control (cells without lens exposure). qPCR was performed in duplicate with cDNA obtained from the pooled samples, and the results are presented as mean ± standard deviation (19). Statistical analysis was performed using one-way ANOVA followed by Dunnett's multiple comparisons test. A *p*-value <0.05 was considered statistically significant (19).

**Table 2:** Sequence of primers for gene expression study.

Target gene	Forward	Reverse
IL-6	5′-GCAGAAAACAACCTGAACCTT-3′	5′-ACCTCAAACTCCAAAAGACCA-3′
IL-1β	5′-CTGTCCTGCGTGTTGAAAGA-3′	5′-TTGGGTAATTTTTGGGATCTACA-3′
TNF-α	5′-GACAAGCCTGTAGCCCATGTTGTA-3′	5′-CAGCCTTGGCCCTTGAAGA-3′

### 2.7 Bacterial growth inhibition assay

The impact of the iron-oxide nanoparticle-coated contact lenses against *S. aureus* and *P. aeruginosa* was evaluated using a bacterial growth inhibition assay based on optical density (OD_600_) measurements. In fresh LB broth, bacterial cultures were diluted overnight to a final concentration of about 1 × 10^6^ CFU mL^−1^ (OD_600_ = 0.05). Sterilized NP-coated lenses and clear commercially available lenses were aseptically transferred into sterile 24-well plates containing 2 mL of bacterial suspension. The experimental setup included a positive control consisting of a bacterial suspension without a lens and a negative control containing sterile broth only. All groups were incubated at 37°C under gentle shaking for 24 h to ensure adequate aeration and uniform bacterial dispersion. The optical density of each culture was measured at 600 nm using a Tecan Infinite plate reader, following incubation and lens removal. The degree of bacterial growth inhibition was calculated by comparing OD_600_ values of NP-coated lenses and clear lenses with those of the positive control using the Equation 2.

$$\mathrm{Percent}\;\mathrm{inhibition}\;=\frac{1-\;\mathrm{ODlens}\;}{\mathrm{ODcontrol}}\times 100$$ (2)

Each test was run in triplicate with three independent wells per group, and the results are reported as mean ± standard deviation. One-way ANOVA was used for statistical analysis, with *p* < 0.05 considered statistically significant (20, 21).

### 2.8 Characterization and analysis

A PAN analytical Empyrean X diffractometer using Cu Kα radiation (*λ* = 1.5418 Å) was used to record the experimental powder X-ray diffraction patterns of the iron-oxide nanoparticle-embedded lenses. A continuous scan between 5° and 60° (2*θ*) with a step size of 0.01° was used to measure the powdered medication in a silica sample holder. FTIR analysis was performed to identify any possible chemical bonding or interactions between the nanoparticles and the functional groups of the lens matrix. A PerkinElmer Spotlight 200 FTIR microscopy system was used to conduct the test analysis. Spectra were recorded with a model spectrometer over the range of 4,000–400 cm^−1^. Two types of samples were analysed: a clear lens without embedded nanoparticles and a lens embedded with iron oxide nanoparticles. X-ray photoelectron spectroscopy was used to confirm the surface elemental composition and chemical states of the iron oxide nanoparticles embedded within the contact lenses. A monochromatic Al Kα (1,486.6 eV) X-ray source under ultrahigh-vacuum conditions was used for the measurements. The analysis was performed using an ESCALAB Xi+ XPS system (Thermo Fisher Scientific). During the analysis, Al Kα radiation was used with a spot size of 650 μm. Avantage software (version V5.9931, Thermo Fisher Scientific) was used to process the survey and further narrow the spectra. This analysis was performed to determine the percentage of iron oxide nanoparticles. Charge correction was performed using the carbon peak at 284.8 eV. Peak fitting was carried out using the Smart (Shirley) background in Avantage software. Spectra were fitted using the Voigt (Convolve) function with an L/G mix of 30%, a tail mix of 100%, and tail height and tail exponent set to 0. The elemental and morphological characterization of lenses was performed using SEM, transmission electron microscope (TEM), and EDX. Surface morphology and nanoparticle distribution were examined using a Phenom XL Desktop SEM (Thermo Fisher Scientific), operated at 5–15 kV. Elemental analysis was carried out using the EDX detector integrated with the SEM. Further imaging was performed using TEM to investigate the surface morphology. The NP size and standard deviations were investigated via a Tecnai TEM operated at 200 kV. The TEM has a resolution of 0.24 nm over a voltage range of 20–200 kV. The optical transmission behaviour of the contact lenses incorporating nanoparticles was studied using a USB 2000+ UV–vis spectrophotometer. The spectrophotometer was capable of measuring wavelengths ranging from 200 to 1,100 nm. Behaviour was studied as a function of nanoparticle loading using two different embedding strategies: (i) repeated cycles of immersion (increasing effective nanoparticle loading in the lens matrix through repeated cycles of dipping in iron solution) and (ii) varying the molarity of Fe^2+^/Fe^3+^ precursor ions used during lens uptake. The resulting transmission vs. wavelength curves were plotted. The energy band gap was calculated through the Tauc plot analysis. UV–visible light spectra were measured in the wavelength range from 400 to 800 nm.

## 3 Results and discussion

The average values of the nanoparticle diameter and interparticle spacing in a unit cell with a rectangular close packing were utilized for electromagnetic modelling of the self-assembled films. Schartz and colleagues demonstrated that abnormalities in the 2D lattice, such as polycrystallinity, slight fluctuations in particle diameter, and interparticle gaps, do not cause significant changes in the optical properties (22). Therefore, the average nanoparticle size is sufficient to represent the optical properties of the nanoparticles in the electromagnetic models.

Because they are rapidly cleared by renal clearance, magnetic nanoparticles smaller than 10 nm are not preferred for some biomedical applications. Furthermore, it has been demonstrated that both the largest and smallest nanoparticles appear to be harmful to cells, causing apoptosis, oxidative stress, DNA damage, and mutagenesis (23, 24).

**Figure 3:**
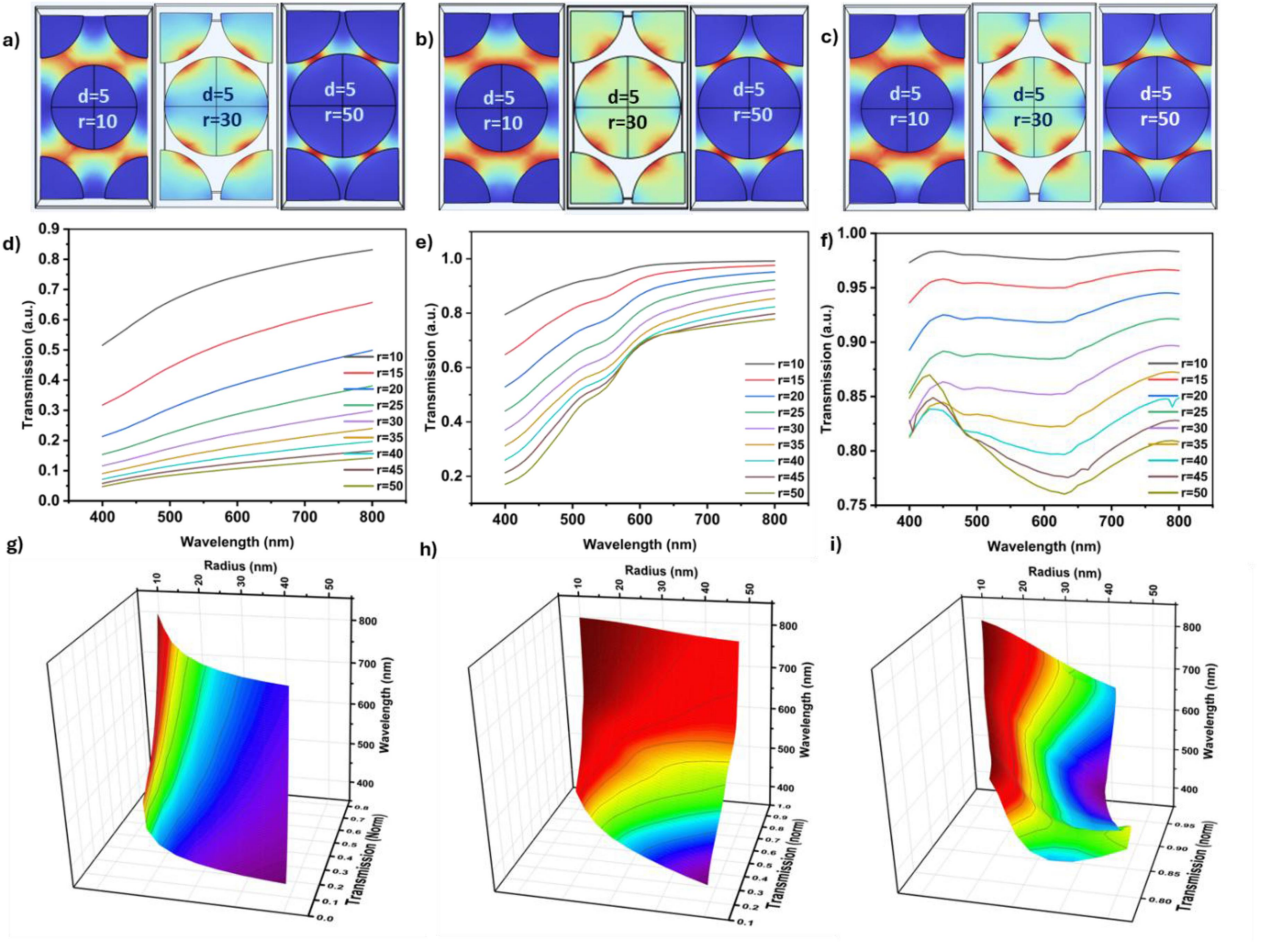
Simulated optical transmission and far-field distributions of Fe, Fe_2_O_3_, and Fe_3_O_4_ nanoparticles dispersed in water medium for different particle radii (10–50 nm). Electric field distributions of **(a)** Fe NPs, **(b)** Fe_2_O_3_ NPs, and **(c)** Fe_3_O_4_ NPs. Transmission spectra of **(d)** Fe NPs, **(e)** Fe_2_O_3_ NPs, and **(f)** Fe_3_O_4_ NPs. 3D plots for **(g)** Fe NPs, **(h)** Fe_2_O_3_ NPs, and **(i)** Fe_3_O_4_ NPs.

Electromagnetic modelling for nanoparticles dispersed in water was performed. Figures 3a–c show the electric field distributions of Fe, Fe_2_O_3_, and Fe_3_O_4_ NPs, respectively. The transmission spectra show a consistent trend for all three materials: as the particle radius increases, the transmission decreases across the visible region (400–800 nm). For subwavelength particles, the absorption cross section scales approximately with *r*^3^, whereas the scattering cross section increases more rapidly, scaling roughly with *r*^3^ in the Rayleigh regime (25). As a result, larger nanoparticles remove more energy from the transmitted beam, reducing the forward transmission intensity. At shorter wavelengths, transmission drops more steeply, particularly below ≈ 500 nm, corresponding to stronger electromagnetic coupling and higher scattering efficiency in that range. This wavelength-dependent extinction behaviour is consistent with previous optical studies on transition-metal and oxide nanoparticles (26, 27)*.*

Each material exhibits different spectral features. Fe_2_O_3_ shows a smoother rise in transmission towards longer wavelengths (Figure 3e), which shows its indirect band gap and lower absorption beyond ≈600 nm (28). Magnetite presents transmission spectra (Figure 3f) with dips near 400–550 nm that evolve with particle radius. These dips are likely associated with localized plasmon-like resonance modes in magnetite due to its higher imaginary and real parts of the refractive index in this range (29). Metallic Fe nanoparticles exhibit the strongest attenuation at short wavelengths and a monotonic rise in transmission with increasing wavelengths (Figure 3d), consistent with strong free-electron absorption and interband damping (16).

The 3D transmission surfaces for Fe, Fe_2_O_3_, and Fe_3_O_4_ NPs are shown in Figures 3g–i, which show the wavelength–radius dependence. The transmission surface declines steeply with increasing particle radius, particularly below 500 nm, confirming the enhanced scattering contribution at shorter wavelengths.

**Figure 4:**
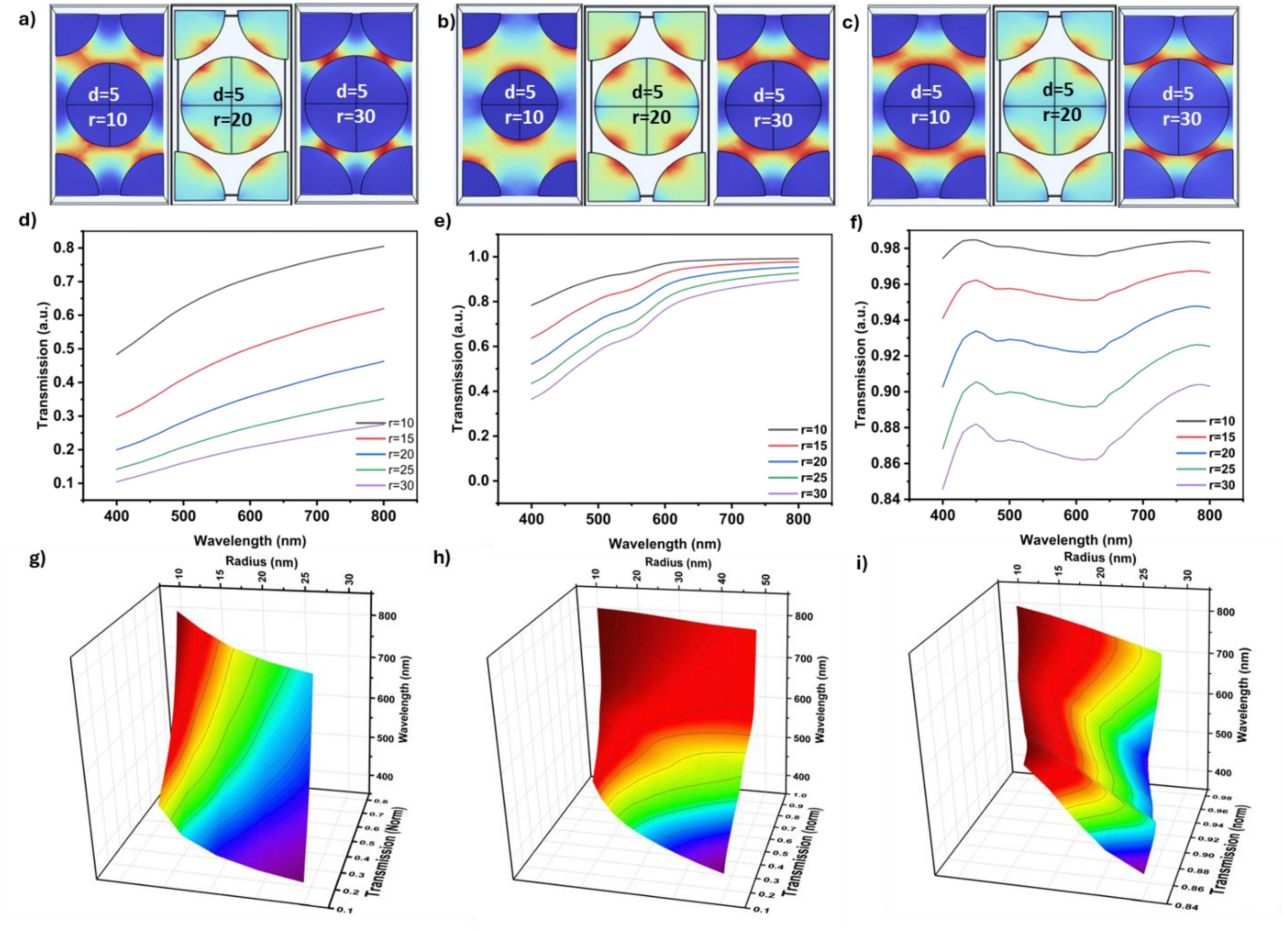
Illustration of the simulated optical transmission and far-field distributions of Fe, Fe_2_O_3_, and Fe_3_O_4_ nanoparticles dispersed in contact lens medium for different particle radii (10–50 nm). Electric field distributions of **(a)** Fe NPs, **(b)** Fe_2_O_3_ NPs, and **(c)** Fe_3_O_4_ NPs. Transmission spectra of **(d)** Fe NPs, **(e)** Fe_2_O_3_ NPs, and **(f)** Fe_3_O_4_ NPs. 3D plots for **(g)** Fe NPs, **(h)** Fe_2_O_3_ NPs, and **(i)** Fe_3_O_4_ NPs.

Electromagentic modelling for iron nanoparticles dispersed in contact lens medium is shown in Figure 4. The overall trend remains consistent with that observed in water—transmission decreases gradually with increasing nanoparticle radius across the visible wavelength range (400–800 nm). However, compared with the aqueous environment, the rate of transmission decay is slightly reduced, and the overall transmission values are higher for the same particle size. A smaller refractive index mismatch leads to weaker scattering and reflection losses, thus preserving higher transmission (27).

These simulations confirm that embedding nanoparticles within the contact lenses enhances optical transparency while maintaining the general size-dependent extinction trend. Results indicate that the smaller nanoparticles, which are less than 15 nm, remain optimal for achieving high transparency, whereas larger nanoparticles still produce noticeable attenuation, particularly in the blue region of the spectrum.

### 3.1 Validation of modelling with Mie scattering theory

**Figure 5:**
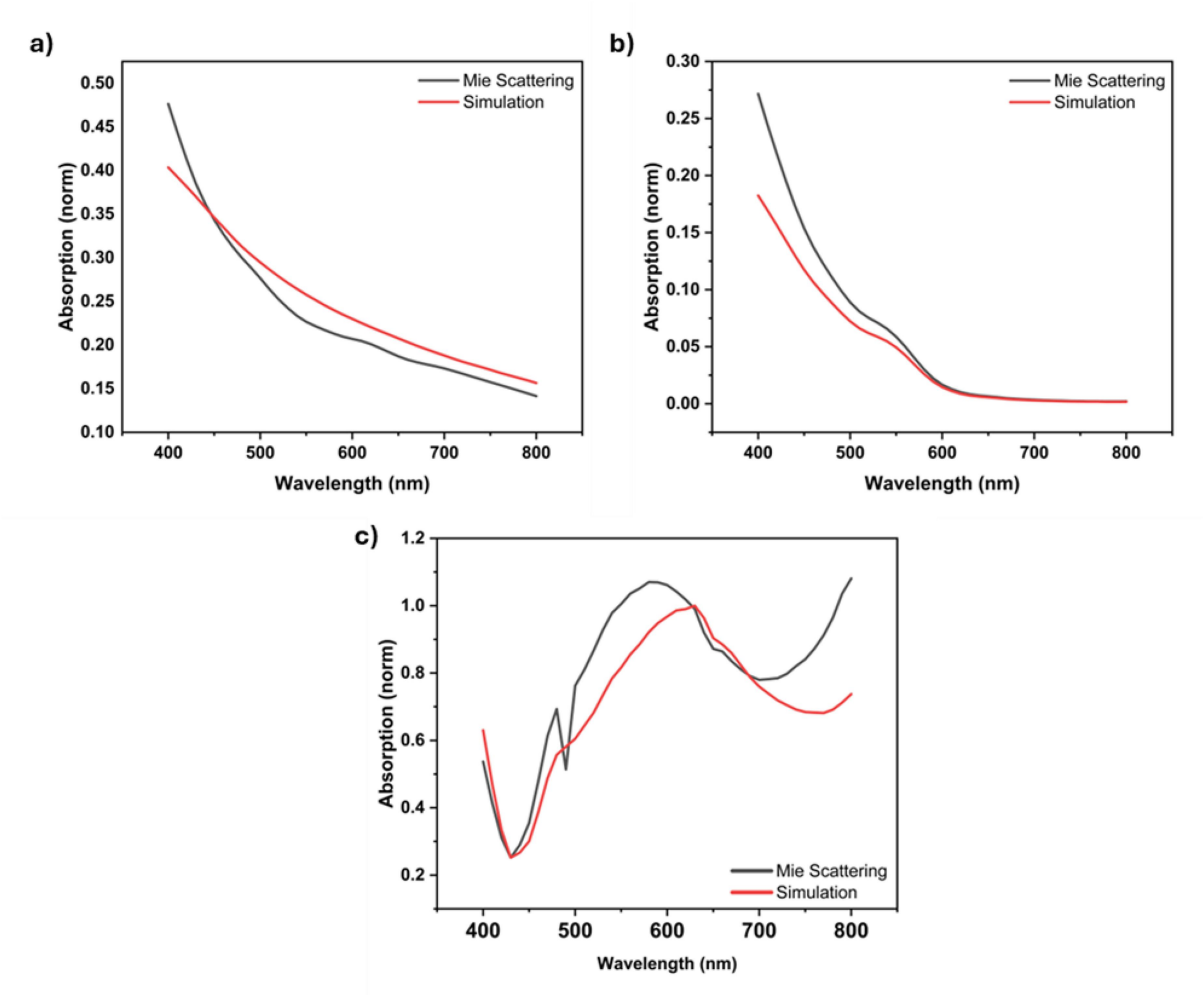
Comparison of absorption with Mie scattering and electromagnetic modelling. **(a)** Fe, **(b)** Fe_2_O_3_, and **(c)** Fe_3_O_4._

The optical absorption behaviour of Fe, Fe_2_O_3_, and Fe_3_O_4_ nanoparticles obtained from simulations was compared using Mie scattering theory. As presented in Figure 5, both methods exhibit comparable trends, confirming the reliability of the simulated approach. For Fe nanoparticles (Figure 5a), absorption decreases gradually with increasing wavelength, characteristic of metallic behaviour dominated by free-electron damping (30).

In the case of Fe_2_O_3_ nanoparticles, both spectra show reduced absorption and a clear decline beyond ∼600 nm (Figure 5b) corresponding to its semiconducting nature and an optical band gap near 2.1 eV (31). Overall, the strong agreement between the two methods validates the computational model and demonstrates the suitability of COMSOL simulations for predicting the response of iron and iron oxide NPs.

**Figure 6:**
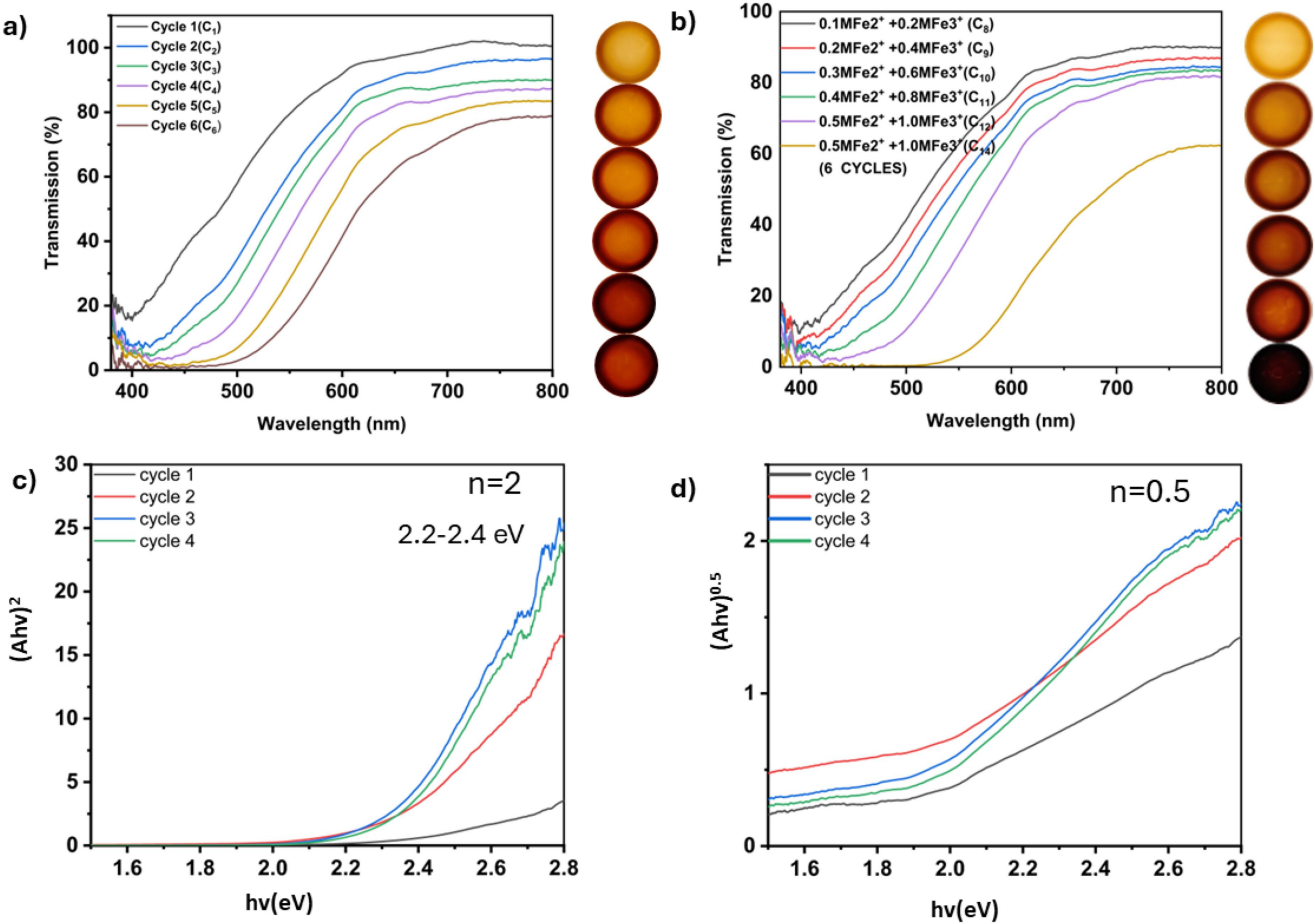
UV-vis spectra of **(a)** contact lenses obtained by incorporating nanoparticles through different cycles of immersion and **(b)** lenses obtained through varying the molarity of Fe^2+^/Fe^3+^ precursor ions during lens uptake. **(c)** Tauc plots for direct transition and **(d)** indirect transition of contact lenses obtained by incorporating nanoparticles through different cycles of immersion.

Optical transmission visibly varies with the concentration of embedded nanoparticles. At lower nanoparticle concentrations (C_1_, C_2_, C_8_, and C_9_, as given in Table 1), the lenses remain highly transparent, with high transmission (approximately 80%–90%) across the 500–800 nm region (Figures 6a,b), whereas at higher NP loading (C_4_, C_5_, C_6_, C_10_, C_11_, C_12_, and C_14_, as given in Table 1), the transmission decreases and the lenses appear visibly darker. The differences in NP concentration among the lenses are provided in Supplementary Figure S2.

The spectral shape also changes broadly. At higher NP loading, stronger attenuation is observed across the visible region, and the onset of strong absorption shifts further into the visible region, indicating that the embedded NPs influence the optical path of the lens matrix. The high transmission in the visible range indicates that the embedded iron oxide nanoparticles do not significantly scatter or absorb visible light, thereby preserving the lens's optical clarity and suitability for vision correction.

Tauc plot analysis was performed to characterize the optical absorption onset of the iron-oxide nanoparticle-embedded commercial lens samples. In this approach, the absorption coefficient was approximated from the absorbance, assuming comparable lens thickness; therefore, it represents an effective absorption coefficient for the composite system. This approach has been widely adopted for nanoparticle-embedded and composite optical systems, where precise thickness-corrected absorption coefficients are experimentally difficult to determine (32, 33). Baseline correction was performed by subtracting the absorbance spectrum of a blank commercial lens, thereby isolating the optical contribution of the embedded iron oxide nanoparticles from the polymer matrix background.

Tauc plots for indirect (*n* = 0.5) and direct (*n* = 2) transitions for samples obtained through different cycles are provided in Figures 6c,d, respectively. It is acknowledged that for mixed Fe_2_O_3_ and Fe_3_O_4_ systems, both direct and indirect optical transitions may contribute to the absorption behaviour, and assigning a single transition character is not always straightforward (34, 35). Fe_2_O_3_ typically exhibits an indirect band gap of ∼1.2–2 eV and direct band gaps at around 1.9–2.7 eV (36, 37), while Fe_3_O_4_ is reported to possess direct and indirect transitions in the range of ∼2.2–2.8 eV (38).

The *n* = 2 plots exhibited a quasi-linear absorption onset in the energy range of approximately 2.2–2.4 eV, while the *n* = 0.5 plots showed a gradual low-energy absorption tail below ∼2.0 eV. Both representations suggest that the optical transitions cannot be conclusively assigned to a purely direct or indirect mechanism and that both may contribute to the overall absorption behaviour. The unit of (Ahv)*^n^* is expressed in arbitrary units, as the absorption coefficient was approximated from the measured absorbance. The constant scaling factor does not affect the extrapolated *x*-intercept and, hence, does not influence the estimated optical absorption onset energy. Tauc plots for lenses prepared with varying NP concentrations are provided in Supplementary Figure S8.

Limitations inherent to this approach, including scattering contributions, matrix absorption, and the subjectivity of linear-region selection, are acknowledged, and the extracted onset values are reported as approximate absorption onset energies for the system.

To check the catalytic efficiency and self-cleaning activity of iron-oxide nanoparticle-embedded contact lenses, a catalytic degradation experiment of methylene blue was conducted. MB is commonly used as a model organic pollutant due to its stable structure, strong absorption in the visible region, and ease of monitoring by spectrophotometric methods (39, 40). Degradation of MB over time was tracked by measuring absorbance at a characteristic wavelength of 666 nm using UV–vis spectroscopy.

**Figure 7:**
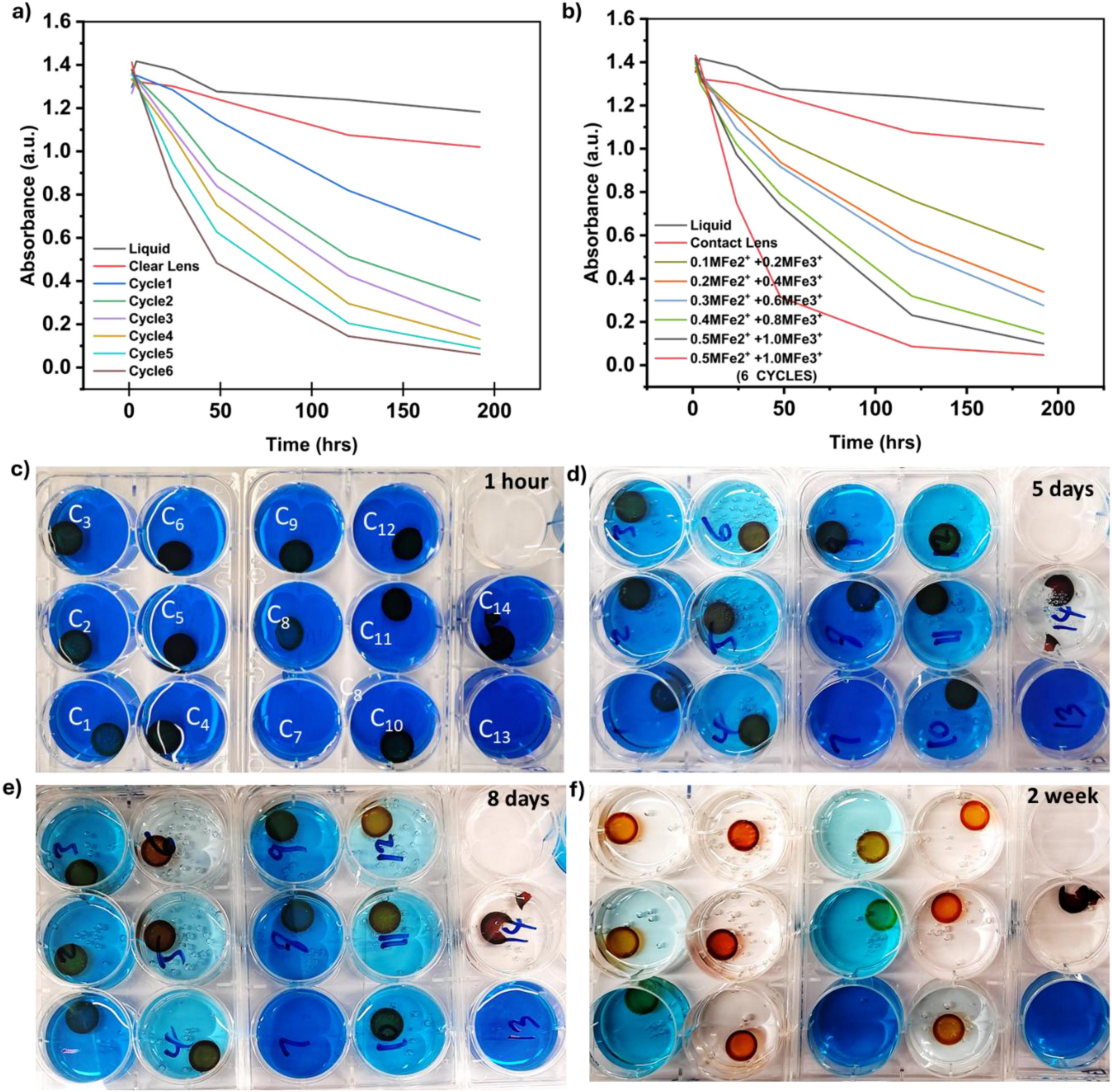
Absorbance spectra for **(a)** contact lenses obtained by incorporating nanoparticles through different cycles of immersion and **(b)** lenses obtained through varying the molarity of Fe^2+/^Fe^3+^ precursor ions. Degradation of methylene blue after: **(c)** 1 h, **(d)** 5 days, **(e)** 8 days, and **(f)** 2 weeks.

Absorption spectra for the contact lenses obtained by incorporating nanoparticles through different cycles of immersion are given in Figure 7a. Absorption spectra for lenses obtained by varying the molarity of Fe^2+^/Fe^3+^ precursor ions are given in Figure 7b. Absorption spectra for the clear lens in MB solution and for MB with H_2_O_2_ alone are included in each figure and also provided in Supplementary Figure S6. The degradation percentage of MB was calculated using the standard formula given in Equation 1. The results showed a clear concentration-dependent trend. The lens material itself did not cause a considerable change in the dye. Methylene blue in the presence of H_2_O_2_ alone showed 12% degradation over 2 weeks. When MB was in contact with lenses loaded with lower concentrations of nanoparticles (C_1_, C_2_, C_8_, and C_9_, as given in Table 1), the absorbance decreased over time, corresponding to 69.8% degradation and indicating modest catalytic activity. In contrast, lenses with higher NP concentration (C_4,_ C_5,_ C_6_, C_10_, C_11_, C_12_, and C_14_, as given in Table 1) showed a faster decrease in absorbance, with 96.7% methylene blue degradation (within 2 weeks) in, resulting in a completely clear solution at the end of 2 weeks (Figures 7c–f). The sample codes corresponding to the NP concentration of each lens dispersed in MB are labelled on the well plate in Figure 7c.

Under peroxide-free conditions, the clear lens exhibited negligible MB removal, confirming that the lens substrate does not interact with the dye. In contrast, the NP-coated lens showed substantial MB removal (∼55% degradation in 4 days), indicating a strong nanoparticle-mediated contribution. The adsorption behaviour of iron oxide nanoparticles is well documented (41). Upon addition of H_2_O_2_, an improvement in degradation efficiency was observed, with the highest removal efficiency and catalytic enhancement associated with the presence of iron oxide nanoparticles (∼77.4% degradation in 4 days). The approximately 22% increase suggests a catalytic contribution in addition to the adsorption-driven removal process. The clear lens in the peroxide condition showed reduced degradation (∼59.1% degradation in 4 days) than the NP-coated lens. Iron oxide nanoparticles have been widely reported to exhibit catalytic activity in MB degradation systems (42). Further studies are needed to clarify the underlying mechanisms of MB removal. In particular, direct detection of ROS, such as hydroxyl radicals and superoxide species, as well as adsorption–desorption studies, would help distinguish catalytic oxidation from surface-mediated dye removal. These findings are further supported by the biocompatibility studies. The corresponding graphs are provided in Supplementary Figure S3.

Thus, the observed colour change from blue to clear over 2 weeks confirms that the iron oxide nanoparticles embedded in the lens exhibit adsorption and catalytic behaviour, suggesting potential applications in optical devices with self-cleaning or antimicrobial functionalities.

**Figure 8:**
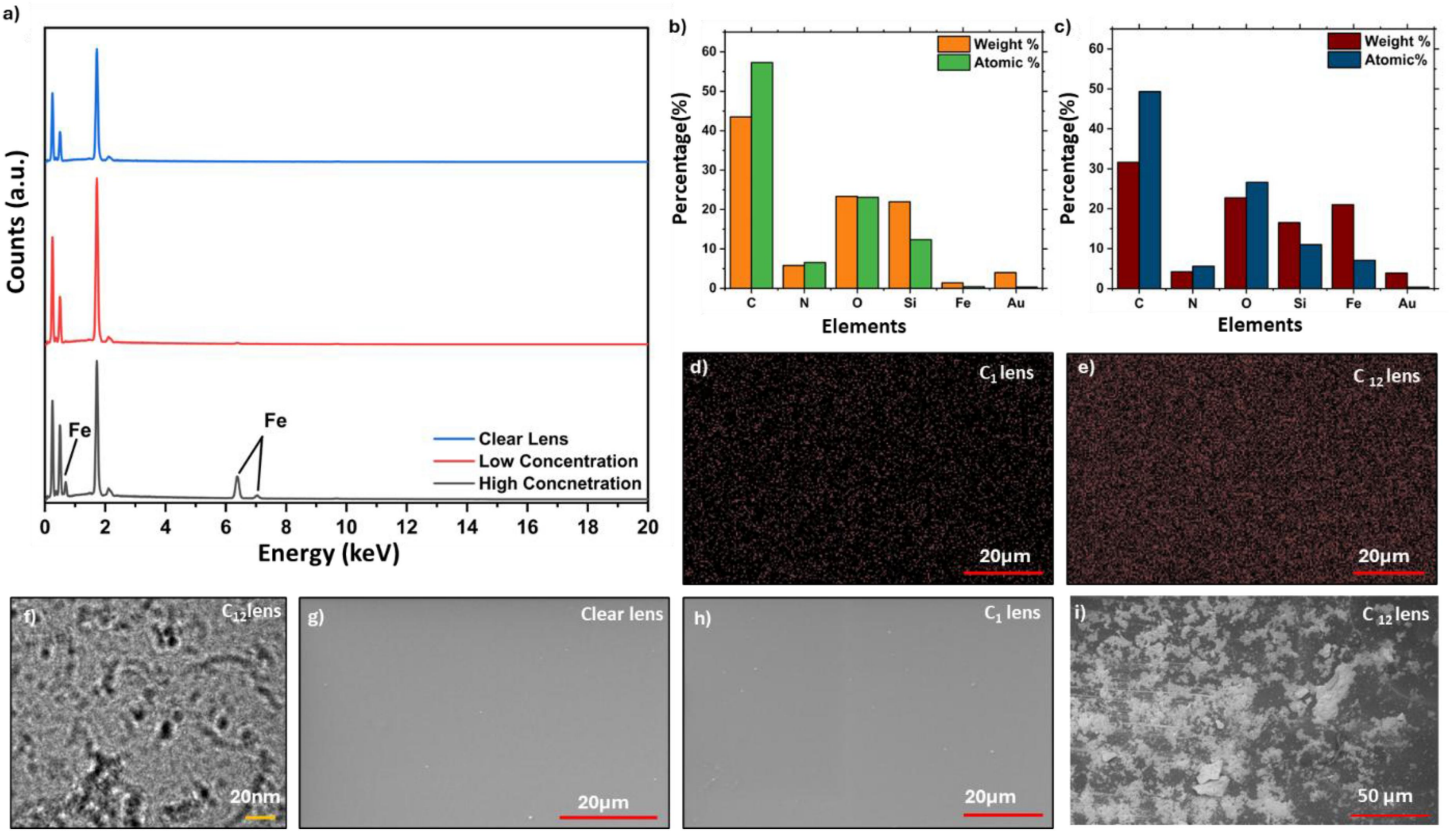
**(a)** EDS spectra. Elemental weight% and atomic% distribution for **(b)** low- (C_1_) and **(c)** high-concentration lenses (C_12_). **(d)** Distribution of iron nanoparticles in the low-concentration lens (C_1_) and **(e)** high-concentration lens (C_12_). **(f)** TEM image of the sample (C_12_). SEM images of the **(g)** clear lens, **(h)** low-concentrated lens (C_1_), and **(i)** high-concentrated lens (C_12_).

Figure 8 shows the results of SEM, TEM, and EDS analyses of iron oxide nanoparticles. EDS analysis of iron-oxide nanoparticle-embedded lenses confirms the successful incorporation of iron oxide nanoparticles into the lens. The spectra for the clear lens show only C < O < N and Si from the lens material, with no detectable Fe peak. In the iron nanoparticle-loaded samples, a clear Fe signal, in addition to the matrix elements, and a higher concentration of oxygen (Figure 8a) demonstrate the presence of iron oxide.

These data support the potential use of NPs in a variety of applications, such as catalysis, magnetic materials, and biological applications, and are useful for understanding the chemical makeup and structural features of the particles (43). Quantitative EDS revealed that the Fe and O weight percentages increase systematically from essentially zero in the pristine lens to a low but measurable value in the loaded lenses, reaching up to 6 wt% and 1.68 at%. The elemental mapping clearly showed the presence of iron with good distribution in the sample.

The TEM image of the nanoparticles revealed roughly spherical particles with an average size of ∼8 nm (Figure 8f). A histogram of particle distribution obtained through TEM image analysis using ImageJ software is provided in Supplementary Figure S5. The SEM images of the clear lens (Figure 8g) did not show the presence of iron. While the low-concentration lenses (Figure 8h) showed minimal NPs, they were observed as isolated bright spots corresponding to nanoparticle clusters (additional SEM images are given in Supplementary Figure S7). At high concentrations, the number of NPs increased without the formation of cracks, indicating that the embedding of iron oxide NPs did not compromise the lens morphology (Figure 8i). Similar results have been reported in the literature (44). Overall, the surface morphologyand microstructure of the produced NPs were clearly characterized through the SEM and TEM images.

While SEM, TEM, and EDS analyses confirmed successful nanoparticle incorporation and apparent uniform distribution within the lens matrix, the present study did not yet establish long-term stability under relevant wear conditions. Especially, the possible nanoparticle leaching was not evaluated. Future studies should, therefore, incubate lenses in simulated tear fluid or PBS for defined periods, followed by ICP-MS or ICP-OES analysis of the incubation medium to quantify released iron and assess safety and durability. In addition, wear-simulation experiments such as stress, blinking-like motion, protein adsorption, and tear film components, which are needed to determine whether prolonged use affects nanoparticle retention or catalytic activity, are intended to be explored in the future.

It should be noted that the experimentally synthesized nanoparticles were identified by XRD and XPS as a mixed Fe_2_O_3_/Fe_3_O_4_ system, whereas modelling was performed for idealized single-phase Fe_2_O_3_ and Fe_3_O_4_ nanoparticles separately. The modelling is intended as an idealized, trend-based analysis of how particle size and the optical properties of iron oxide influence transmission in the lens environment.

**Figure 9:**
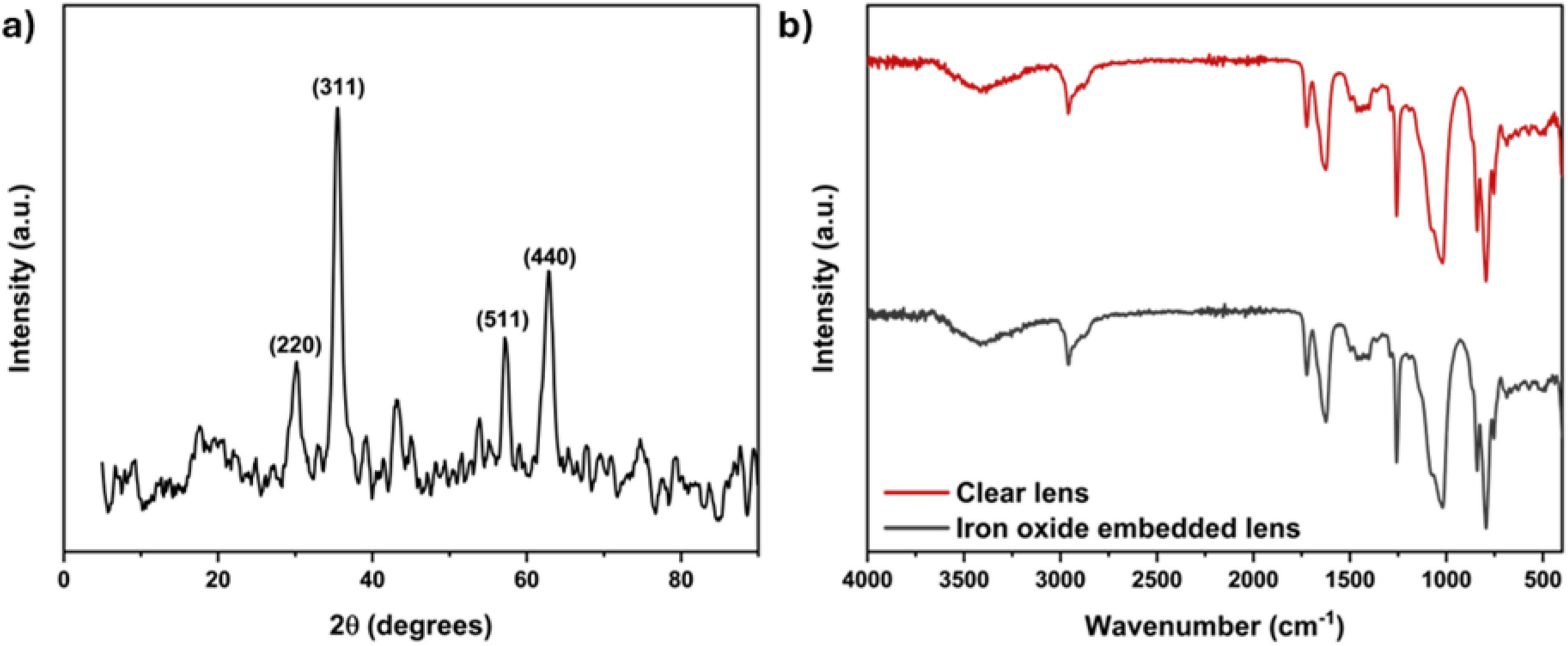
**(a)** X-ray diffraction (XRD) pattern of iron oxide nanoparticle-embedded contact lens (C_12_). **(b)** FTIR spectra for the clear lens and iron nanoparticle-embedded lens (C_12_).

The XRD diffraction peaks of the nanoparticles generated exhibit the diffraction peaks matching well with the cubic spinel structure typical of magnetite (Fe_3_O_4_, JCPDS 19-0629) and maghemite (*γ*-Fe_2_O_3_, JCPDS 39-1346). As summarized in Table 3, the peak positions of our sample lie slightly between those of the two reference phases, indicating a partial or mixed oxidized structure (Figure 9a). In addition, the calculated lattice parameter of the synthesized sample was found to be 8.37 Å, which lies intermediate between the standard values for magnetite (8.396 Å) and maghemite (*a* = 8.33–8.36 Å). We can say that the structures of magnetite and maghemite are similar but not identical. The specific reason is that the bulk *γ*-Fe_2_O_3_ structure shows the presence of vacancies, while there are both tetrahedral sites fully occupied with Fe(III) spin states and octahedral sites fully occupied with Fe(II) and Fe(III) spin states in the Fe_3_O_4_ phase (45).

**Table 3:** Comparisons of XRD data for magnetite, maghemite, and the sample formed.

Sample	JCPDS file number	Angle 2*θ* (degrees)			
		(220)	(311)	(511)	(440)
Standard magnetite	39-1346	30.1	35.4	56.9	62.5
Standard maghemite	19-0629	30.3	35.7	57.3	63
Present sample		30.06	35.52	57.25	62.92

Similar observations, in which it is not easy to distinguish *γ*-Fe_2_O_3_ from Fe_3_O_4_ using XRD patterns because of their similar structures, have been reported in the literature (43, 46, 47).

The FTIR spectra for clear, low-concentration, and high-concentration nanoparticle-embedded lenses (C_12_) are shown in Figure 9b. The FTIR of the synthesized iron oxide nanoparticles was recorded over the wavenumber range of 400–4,000 cm^−1^. All three spectra exhibit similar characteristic transmission bands. No additional peaks, significant shifts, or broadening of existing peaks were observed in the nanoparticle-loaded samples compared with the clear lens. The band at ∼3,400 cm^−1^ indicates the O–H stretching in OH^−^ groups. The absorption peak at around 1,604 cm^−1^ showed asymmetric and symmetric bending vibrations of C=O, and the band at 1,467 cm^−1^ is attributed to C–H bending. The absorption peak at 1,620 cm^−1^ can be attributed to C=O groups. The 1,261, 1,721, and 1,625 cm^−1^ peaks can be ascribed to the C–N stretch, C=O stretch, and C=C stretch, respectively (19). This suggests that the incorporation of nanoparticles did not result in a strong bonding or any kind of modification of the polymer's chemical structure. The absence of new peaks confirms that the interaction between the NP and lens matrix is physical rather than chemical. This finding indicates that the hydrogel chemistry remains unaltered and that wettability remains the same. A lack of shift or new group formation was reported in another study by Hisham et al. (48). FTIR spectra of polymer composites containing Fe_2_O_3_ or other metal oxides often show no new transmission bands because the nanoparticles of magnetic chitosan nanocomposites mainly act as fillers or reinforcing agents rather than forming chemical bonds. Therefore, the FTIR results confirm the successful embedding of nanoparticles within the lens matrix without compromising the polymer's chemical integrity, which is a desirable outcome for maintaining the biocompatibility and optical clarity.

**Figure 10:**
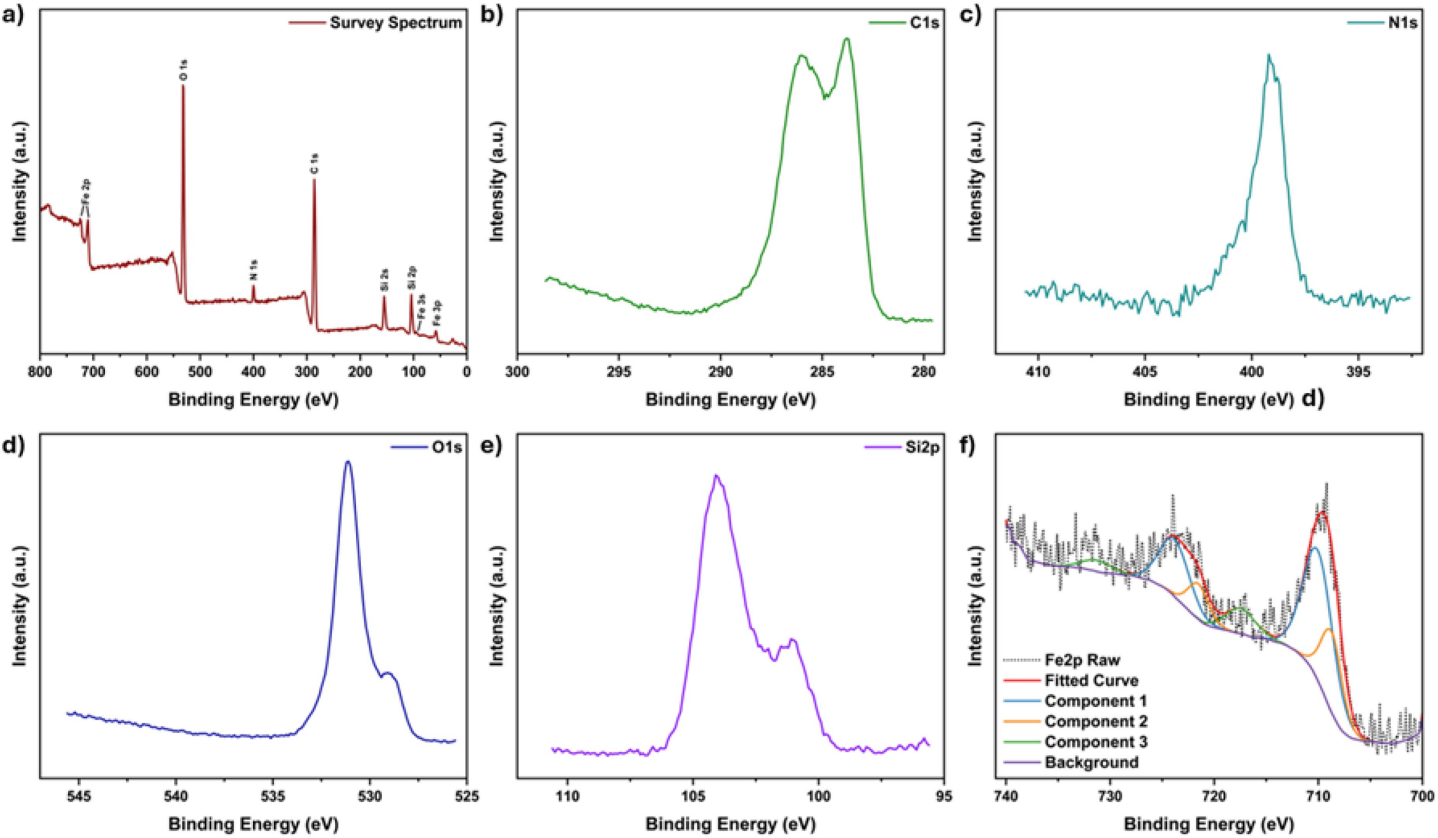
**(a)** XPS survey spectra for iron oxide lens. XPS high-resolution deconvolution of **(b)** 1s region of carbon, **(c)** 1s region of nitrogen, **(d)** 1s region of oxygen, **(e)** 2p region of silicon, and **(f)** 2p region of iron.

The high-resolution XPS spectrum of Fe 2p (Figure 10) shows the presence of Fe^2+^ and Fe^3+^ oxidation states, indicating that the composition is a mix of Fe_3_O_4_ and Fe_2_O_3_. The Fe 2p_3/2_ region shows two main peaks at 709.5 and 710.8 eV, corresponding to Fe^2+^ and Fe^3+^, respectively. The Fe 2p_1/2_ region shows peaks at 722.3 and 724.6 eV, with shake-up satellites at ∼732.1 and ∼718.3 eV. The spin–orbit splitting of about 13.8 eV and the strong satellite intensity show that the iron is oxidized, not metallic. Quantitative fitting shows that the ratio of Fe^3+^ to Fe^2+^ areas is about 2.3:1, which is a little higher than the stoichiometric 2:1 that would be expected for pure magnetite (Fe_3_O_4_). This suggests the possible presence of maghemite (*γ*-Fe_2_O_3_). The spectral characteristics collectively indicate that the iron oxide sample comprises both Fe_3_O_4_ and *γ*-Fe_2_O_3_. The oxygen high-resolution scan shows a secondary peak at 529.1 eV corresponding to the oxygen from the metal oxide. XPS survey spectra and high-resolution spectra of the clear lens are given in Supplementary Figure S1.

Although the XRD and XPS results are consistent with a mixed magnetite/maghemite composition, distinguishing between the two remains challenging due to their closely related crystal structures and overlapping spectral features. Therefore, future studies could employ complementary techniques, such as Mössbauer or Raman spectroscopy, to achieve more precise phase detection.

Contact angle measures the hydrophilic nature, or wettability, of the hydrogel surface. In the present study, NP-embedded lenses were synthesized using two methods. The contact angle of the lenses fabricated by changing concentration is 84.3°, for the lenses obtained by repeating cycles is 84.4°, and for the clear lens is 84.96° (images are provided in Supplementary Figure S4). A contact angle below 90° indicates hydrophilic lenses (49). The relatively higher contact angle observed may be attributed to surface modifications introduced by the nanoaprticle embedding. The measured contact angle suggests that the lens maintains acceptable wettability, comparable to that of commercial materials.

### 3.2 Biocompatibility analysis

**Figure 11:**
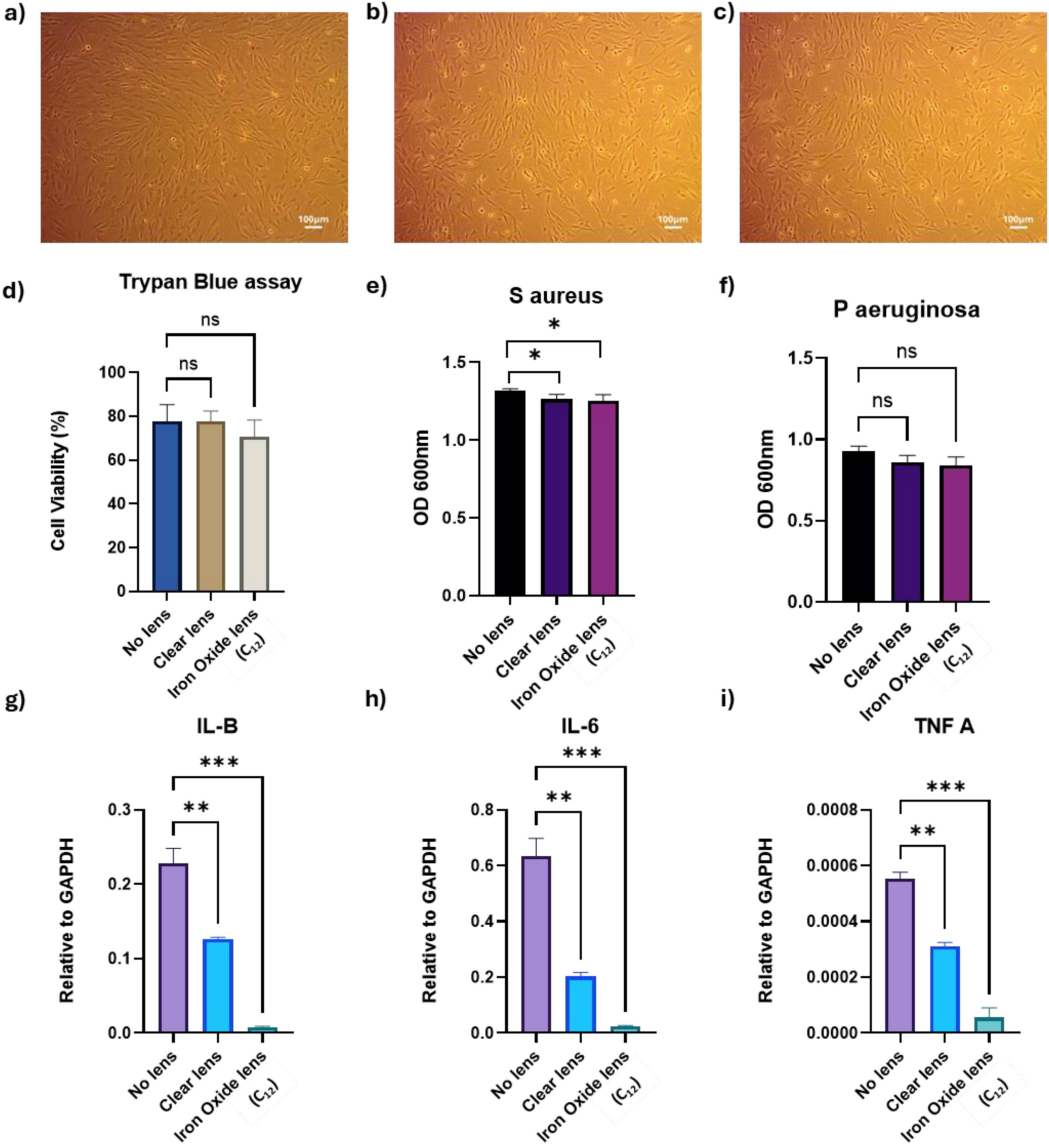
Microscopy images of cells exposed to **(a)** no lens, **(b)** clear lens, and **(c)** NP-embedded lens (C_12_). **(d)** Cell viability assay. **(e,f)** Antibacterial assay. **(g–i)** Anti-inflammatory assay.

The inverted light microscopy images of cells after 24-h incubation with the prepared iron oxide lens, clear lens, and those of cells without any lens exposure are shown in Figures 11a–c. The cells do not appear to show any visual morphological changes, with or without the lens, indicating good maintenance of cell attachment, growth, and expansion despite lens exposure. However, no quantitative morphometric analysis or high-magnification imaging was performed. The cytocompatibility of nanoparticle-integrated contact lenses was evaluated using the trypan blue exclusion assay on HDFn cells. Figure 11d shows that the viability of cells exposed to NP-coated lenses (C12) remains high, comparable to that of the clear commercial lens and the control group without any lens. The statistical analysis using one-way ANOVA indicates that there is no significant difference (*p* > 0.05) among the three groups, confirming that NPs’ incorporation did not induce measurable cytotoxicity. These results indicate that the lenses maintain good biocompatibility, with cell viability well above the acceptance threshold of 70%. These findings suggest nanostructures are chemically stable and do not release toxic byproducts into the culture medium over the 24-h incubation period.

The relative mRNA expression levels of the pro-inflammatory cytokines IL-1β and IL-6 were quantified by qPCR after 24 h exposure (Figure 11g–i) to determine whether iron oxide nanoparticle integration caused inflammatory signalling. The Ct values were within acceptable limits, and qPCR reactions were performed with appropriate controls. qPCR analysis demonstrated that NP-coated lenses meaningfully suppressed IL-6, IL-1β, and TNF-α expression relative to both the no-lens and clear lens groups. The NP-loaded lens showed the lowest cytokine levels among all conditions. Specifically, IL-6 and IL-1β expression in NP-embedded-lens-treated cells remained close to baseline, showing >80% reductions relative to the no-lens group. NP coating downregulates the inflammatory response in fibroblasts, indicating effective anti-inflammatory activity. The suppression of cytokine induction further supports its suitability for extended ocular contact applications. Although the above tests with human dermal fibroblasts provide a preliminary indication of biocompatibility, further testing with corneal/conjunctival epithelial cells in future work will enhance the robustness of these data.

The antibacterial performance against *S. aureus* (Gram-positive) and *P. aeruginosa* (Gram-negative) was assessed for NP-coated contact lenses (C_12_) by monitoring bacterial growth in liquid culture via optical density (OD_600_) measurements (Figures 11e,f). In these bacterial models, a reduction in OD values was observed in cultures exposed to NP-integrated lenses compared with the control without a lens. For *S. aureus*, the NP-loaded lenses exhibited the most pronounced and statistically significant antibacterial effect relative to the control, whereas the reduction against *P. aeruginosa* was somewhat moderate. The lower optical density values indicate inhibited bacterial proliferation in the presence of NP coating, indicating that the incorporated nanoparticles impart bacteriostatic activity. The results confirm that the incorporation of iron oxide nanoparticles enhances the antibacterial activity of the lenses without compromising their biocompatibility, supporting their potential for infection-resistant ocular applications.

This study provides a preliminary assessment of cytocompatibility and antibacterial activity, with certain limitations. The trypan blue assay evaluates membrane integrity, but not cellular metabolic activity, and OD_600_ measurements reflect turbidity rather than accounting for surface adhesion effects. In addition, only a single time point (24 h) was evaluated. Future studies should incorporate metabolic assays, CFU quantification, viable staining, and time-dependent analyses to better understand cell responses and antibacterial mechanisms.

A critical translational concern for NP-integrated contact lenses is the potential for iron ion leakage and subsequent ROS generation at the ocular surface. Under psychological stress conditions, bare NPs are susceptible to oxidative dissolution, DNA strand breaks, and apoptosis in corneal epithelial cells. To address these risks, several design-level mitigation strategies have been developed. Surface passivation through silica or gold shell encapsulation has been shown to substantially suppress Fe leaching and attenuate surface redox catalysis (50). The simultaneous incorporation of antioxidant agents, such as vitamin E, which is already used in commercial silicone hydrogel lenses, and catalytically regenerative nanoceria, into the hydrogel matrix offers an additional *in situ* ROS buffering mechanism (51). Finally, controlled NP loading through pre-encapsulation in polymeric carriers prior to hydrogel embedding, combined with optimization of cross-linking density to physically entrap particles, has been demonstrated to reduce Fe release and limit nanoparticle migration towards the corneal interface (52). Taken together, these strategies, including surface coating, antioxidant co-loading, and controlled matrix incorporation, form a safety-by-design framework that should be considered an essential checkpoint in the translational development of NP loading in ocular devices.

## 4 Conclusion

In the study, prior to the experimental work, electromagnetic modelling of Fe, Fe_3_O_4_, and Fe_2_O_3_ NPs is performed using COMSOL Multiphysics software. The transmission graph for the Fe_2_O_3_ NPs obtained via simulation closely matched the UV transmission spectra obtained experimentally. Iron oxide (Fe_3_O_4_/*γ*-Fe_2_O_3_) nanoparticles were successfully embedded into commercial contact lenses and characterized using complementary structural, optical, and microscopic techniques. The incorporation has proved highly effective, yielding lenses with excellent transparency, strong catalytic activity, and stable nanoparticle dispersion. UV–vis spectroscopy showed a transmission of nearly 98%, but nanoparticle-loaded lenses showed a controlled reduction in transmission with increasing concentration. Both experimental and simulation spectra were in strong agreement, clearly supporting the trend that higher nanoparticle radius and loading lead to reduced optical transmittance. Tauc plots revealed an optical band gap of approximately 2.35 eV.

XRD analysis validated the iron oxide nanoparticle as having a cubic spinel structure, with diffraction peaks matching the standard JCPDS values of magnetite/maghemite. The calculated lattice parameter (∼8.37 Å) is in close agreement with the reference data. XPS confirms that the NPs formed could be both Fe_3_O_4_/*γ*-Fe_2_O_3_ NPs. SEM imaging revealed the lens surface to be smooth and the presence of nanoparticles. EDS spectra and elemental mapping demonstrated uniform Fe distribution for both low and high concentrations. TEM analysis reveals a spherical structure for the nanoparticles, with an average diameter of 15 nm. Catalytic performance studies showed strong ROS-generating activity of iron oxide nanoparticles. They achieved up to 96% degradation of methylene blue within 2 weeks, showing significant catalytic potential. Along with these results, NPs showed very good biocompatibility under the tested conditions, maintaining fibroblast cell viability well above 70% with no observable morphological changes and indicating suppression of bacterial growth, anti-inflammatory, and antibacterial behaviour against *S. aureus*.

Overall, this work provides a detailed experimental and simulation-based analysis demonstrating that iron oxide nanoparticles can be efficiently integrated into commercial lenses without compromising the optical performance. The combined optical, structural, catalytic, and biological results provide a strong foundation for the development of multifunctional smart lenses with potential applications in ocular therapeutics, controlled drug release, biosensing, and real-time diagnostics. In addition, machine-learning-driven optimization of nanoparticle geometry and distribution may further improve lens performance.

## Data Availability

The original contributions presented in the study are included in the article/Supplementary Material, further inquiries can be directed to the corresponding author.
