# Supplementary material for: Iron-oxide nanoparticle-integrated soft contact lenses: optical optimization, catalytic functionality, and biocompatibility
Source: Front Med Technol. 2026 Jul 24;8:1782878. doi: 10.3389/fmedt.2026.1782878 (PMC13447371; doi:10.3389/fmedt.2026.1782878)
Supplement: Supplementary file 1 [file Datasheet1.docx]

**Supporting Information**

**Iron-Oxide Nanoparticle–Integrated Soft Contact Lenses: Optical Optimization, Catalytic Functionality, and Biocompatibility**

**Iron Oxide nanoparticles integrated lens Synthesis**

**Table S1:** Method 1 Multiple Immersion cycles

| Concentration of Precursor Solutions | Reaction time | NaOH Concentration | Reaction time in NaOH |
| --- | --- | --- | --- |
| 0.1M Fe^2+^ + 0.2M Fe^3+^  ^(^C_1_, C_2,_C_3,_ C_4_ ,C_5_ , C_6_ ) | 30 Mins | 0.5M | inutes |

**Table S2:** Method 2 Variation in precursor concentration.

| Concentration of Precursor Solutions | Reaction time | NaOH Concentration | Reaction time in NaOH |
| --- | --- | --- | --- |
| 0.1M Fe^2+^ + 0.2M Fe^3+^ (C_8_) | 30 Mins |  |  |
| 0.2M Fe^2+^ + 0.4M Fe^3+^ (C_9_) | 30 Mins | 0.5M | 30 Minutes |
| 0.3M Fe^2+^ + 0.6M Fe^3+^ (C_11_) | 30 Mins |  |  |
| 0.4M Fe^2+^ + 0.8M Fe^3+^ (C_12_) | 30 Mins |  |  |
| 0.5M Fe^2+^ + 1.0M Fe^3+^ (C_14_) | 30 Mins |  |  |

**Figure S1:** a) XPS survey spectra and high-resolution spectra of clear lens

**Figure S2:** C_1_ and C_4_ samples were obtained through the cyclic repetition of nanoparticle loading, and C_9_ and C_11_ were obtained through the method of varying the precursor concentrations.

**a)**

**d)**

**c)**

**b)**

**Figure S3:** Comparison of methylene blue (MB) removal under different conditions after 4 days: (a) clear lens in MB solution (in water alone) (b) nanoparticle-coated lens in Mb solution (in water alone) (c) clear lens in MB solution (H₂O₂) and (d) nanoparticle-coated lens in MB solution (H₂O₂).

Figure S4: Contact angle measured for a-c) contact lenses obtained by incorporating nanoparticles through different cycles of immersion. d-f) lenses obtained through varying the molarity of Fe^2+^/Fe^3+^ precursor ions during lens uptake. g-i) Clear lens.

Figure S5: Histogram for particle distribution using TEM.

.

Figure S6: control curve MB + H2O2 (no lens) to show baseline photolytic/chemical loss

Figure S7: SEM images at higher magnification.

Figure S8: Tauc plots for a) direct transition and b) indirect transition for lenses obtained through varying the molarity of Fe^2+^/Fe^3+^ precursor ions during lens uptake.

**XPS Fe2p fitting details**

Figure S9: Fitting data of Fe2p peaks done in Avantage software.
